# Who needs and continues to need paediatric palliative care? An evaluation of utility and feasibility of the Paediatric Palliative Screening scale (PaPaS)

**DOI:** 10.1186/s12904-020-0524-4

**Published:** 2020-02-10

**Authors:** Poh Heng Chong, Janice Soo, Zhi Zheng Yeo, Raymond Qishun Ang, Celene Ting

**Affiliations:** 1 HCA Hospice Care, 705 Serangoon Road, #03-01 Block A @ Kwong Wai Shiu Hospital, Singapore, 328127 Singapore; 2 Ministry of Health Holdings, Singapore, Singapore

**Keywords:** Paediatric palliative care, Home hospice, Needs assessment, Screening tool, Admission

## Abstract

**Background:**

While the populations of children who can benefit from paediatric palliative care (PPC) have been broadly defined, identifying individual patients to receive PPC has been problematic in practice. The Paediatric Palliative Screening scale (PaPaS) is a multi-dimensional tool that assesses palliative care needs in children and families to facilitate timely referrals. This study evaluates its use to manage new referrals and ongoing review of patients receiving home-based PPC in Singapore.

**Methods:**

Using a retrospective cohort study design, 199 patients admitted to receive PPC via clinician screening were scored using PaPaS. Eighty-four patients in two groups were scored again at one of two following milestones: one-year service continuation mark or point of discharge before a year. Accuracy measures were compared against clinical assessment.

**Results:**

96.98% of patients scored 15 and above on admission (indicating need for PPC). Patients assessed at following milestones were effectively stratified; those who continued to receive service after 1 year scored significantly higher (M = 19.23) compared to those who were discharged within a year (M = 7.86). Sensitivity and specificity for PaPaS were calculated at 82.54 and 100% respectively. Overall congruence with clinician-based decisions supports the utility of PaPaS as a screening tool in PPC. Recommendations to improve the scale further are proposed.

**Conclusion:**

The PaPaS is a practical screening tool that signposts PPC needs within the clinical setting. This facilitates early referrals to PPC, without having to specify individual prognoses that are often uncertain. Other benefits include optimised continuity of care and implications for resource allocation.

## Background

As part of a growing movement to improve the care of seriously-ill children, Paediatric Palliative Care (PPC) services around the world face a myriad of challenges. Other than securing sustainable funding [1, 2], efforts to promote service access, both in the patient community and among healthcare providers, have been described [3–5]. In addition, at service transition after a referral to PPC has been made, good communication between healthcare professionals is critical [6], not only for access, but also continuity of care and patient safety [7].

While ethos and approaches are similar, the scope of work in adult and paediatric palliative care can differ [8]. In contrast to most life-threatening illness such as cancers in the adult-setting, both children with life-threatening and life-limiting illness (henceforth combined under ‘life-shortening illness’) present a wide variety of diagnoses. These are broadly categorised into four typologies that include congenital anomalies, metabolic diseases, neurological conditions and conditions that are non-progressive, like cerebral palsy [9]. In fact, the cohort with oncological diagnoses number just around 20–30% [10, 11] in most PPC patient census.

Average length of service in PPC is generally longer than that in adult palliative care [12]. Disease trajectories are often uncertain and can differ widely, even between children with the same diagnosis; individual survival can vary from hours to more than 20 years [13, 14]. Heterogeneity in qualifying medical conditions and overall longer survivals are hallmarks of PPC that together increase the complexity in service planning and implementation [15].

Given all the above considerations, eligibility for PPC is necessarily needs-based rather than prognosis-driven; and an objective tool that clearly defines the patient or family with PPC needs is indicated [16]. In addition to accounting for complex needs and longer length of service, such a tool can help address concerns about service sustainability by providing justification for allocating scarce resources between children with varying needs. Finally, a structured and standardised approach to case referrals can also improve communication between providers at point of referral or handover.

Internationally, the populations of children who can benefit from PPC have been defined [17, 18]. Yet, referrals are often made late or not at all [19]. Though the ‘surprise question’ used effectively in the adult setting has been found to be equally sensitive as a prognostic tool in children [20], it is still inadequate as a screening tool within the PPC ethos of holistic family-centred care that starts from diagnosis.

The Spectrum of Children’s Palliative Care Needs has been conceived to incorporate exactly these multi-dimensional elements, grouping children who are eligible for PPC into five prognostic-based categories with distinct care needs [21], though these needs have not been explicitly specified. This framework remains problematic, with participants in an early validation exercise commenting that categorisation still needed their own consolidated assessments that included: clinical symptoms, treatment outcomes, dependency indicators, psychosocial factors, and patient/carer priorities.

One promising tool for screening patients for PPC needs is the Paediatric Palliative Screening scale (PaPaS) [22]. The PaPaS targets timely referrals to PPC through identifying (screening for) children with palliative care needs. It consists of a series of questions in five domains, which are almost the same domains described in the Spectrum of Needs framework [21]. Each question is weighted and assigned a score depending on the response [23]. The total score is then used to stratify patients into different courses of action; a score of 15 and above indicates that PPC could be initiated [22, 23].

Other than the original authors who had conceived and validated the PaPaS, there have not been published reports of its application in other settings. Prior to its implementation in local policy and practice, we performed an evaluation of the utility and feasibility of the PaPaS as an admission-screening tool within a home-based PPC service. Besides admission, we also explored its use in relation to continuation of PPC, such as reassessment after one year. Our findings, experience and recommendations are reported in this paper.

The primary objective of this study is to assess the utility and feasibility of PaPaS as a referral screening tool to identify paediatric patients who may require PPC. The secondary objective evaluates the utility of PaPaS to determine continuation of PPC after one year.

## Methods

### Design

A non-interventional, retrospective cohort study design was adopted, using data collected as part of standard service provision. For the primary objective, action plans recommended by the PaPaS were compared against those by clinician assessment, the common standard to determine patient admissions. For the secondary objective, we hypothesise that PaPaS can distinguish between patients suitable for interim discharge and those who require continuation of PPC beyond the first year. To this end, two groups of patients—those who continued to received PPC after one year and those discharged within a year—were assessed using the PaPaS a second time based on their respective end-points, and their scores compared.

### Setting

The patient population studied received home-based PPC from a specialist paediatric palliative care service in Singapore. The service is nested within the nation’s largest home hospice service, which provides palliative and supportive care for patients at home. A team of eight multi-disciplinary healthcare providers is dedicated solely to the care of paediatric patients.

All patients admitted have life-shortening conditions as described by Association for Children’s Palliative Care and the Royal College of Paediatric and Child Health [9]. Like similar services elsewhere [6, 11], the team serves a majority of young persons with non-cancer diagnoses (80% of cases). New referrals come mainly from tertiary children hospitals that are government funded, with occasional referrals from private paediatricians or oncologists.

Pre-PaPaS, referrals were screened for admission based on the clinical judgement of a single physician trained in paediatric palliative medicine. As part of normal workflow, patients were also reviewed periodically afterwards at multi-disciplinary team (MDT) meetings, for service continuation or interim discharge.

### Data collection

The study included patients admitted into the service between Apr 2012 and July 2016. No age limits were set. Data was extracted from electronic records in the team’s patient management system.

Scoring was determined by JS, who reviewed patient records at the appropriate time-points, and chose the responses that best fitted descriptions in the records. Information relevant to the PaPaS were never explicitly requested from referring sources before. Hence, a flexible approach to assigning scores was adopted; for each patient, JS studied multiple sources of information (e.g. referral forms, discharge summaries from referring institutions, and notes from internal medical records) to impute scores. The scores were corroborated by PHC, who scored a sub-sample of 20 randomly selected patients (10% of sample) to ensure agreement. Where information required for scoring items in the PaPaS was insufficient or missing, these patients were excluded from analysis. A referral form may have no mention of ‘symptom intensity’ or ‘difficulty of symptom control’, which prevents scoring of one item in the original scale [22]. This patient for example would be excluded from our analysis.

For the second assessment, two groups of patients were identified within the sample: patients who received PPC for more than 1 year, and patients who were discharged before 1 year. PaPaS scores were imputed a second time for individual patients using information either at the one-year mark or at point of discharge. Patients not belonging to either groups were excluded. Sensitivity and specificity values for the screening scale at these subsequent milestones were determined.

All scores were tabulated using Microsoft Excel 2016; statistical analyses (e.g. Student t-test and Chi-squared tests) and presentation of distributions (box-plots) were done with STATA 15. The STARD guidelines is referenced in structuring this report [24].

## Results

**Fig. 1:**
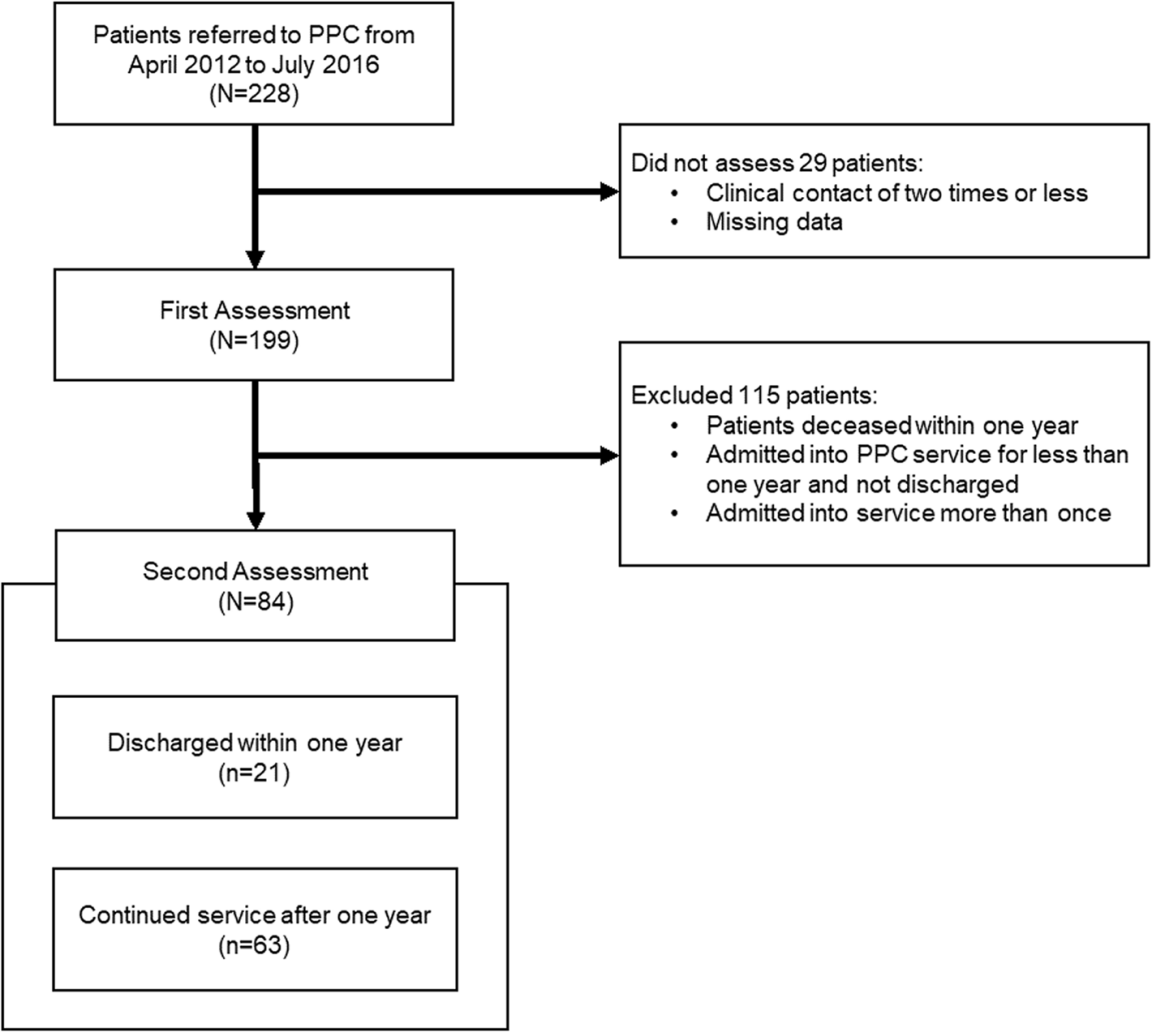
Study population flowchart

Figure 1 details the flowchart for the study population. Two hundred twenty-eight patients were referred for PPC at home between April 2012 and July 2016. Twenty-nine patients (12.71%) were excluded due to insufficient or missing information; the First Assessment scored 199 patients (at point of referral). Eighty-four patients were included in the Second Assessment involving two groups; 115 patients were excluded for various reasons (listed in Fig. 1).

### First assessment

**Fig. 2:**
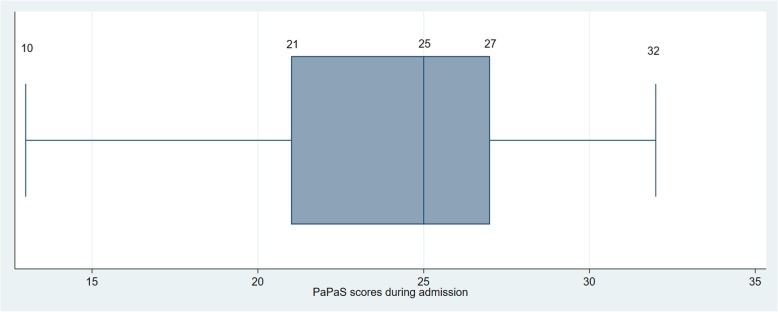
Boxplot distribution of PaPaS scores for First Assessment (*N* = 199)

Table 1 summarises the demographics of patients at First Assessment. Figure 2 shows the box-plot distribution of PaPaS scores for the First Assessment. Scores ranged from 10 to 32 points, with a mean score of 23.71 (*SD*: 4.43; Median score 25). One hundred ninety-three patients (96.98%) scored 15 and above.

**Table 1:** Demographics of patients at First Assessment (*N* = 199)

Demographics	
**Age at referral**	
Median age in years (IQR)	12.6 (12.2)
Range of ages in years	0 (1 day old) to 39.6
Number of patients less than 1 year old	18
Number of patients between 1 and 19 years old	141
Number of patients 19 years old or above	40
**Gender**	
Female (%)	84 (42.2%)
Male (%)	115 (57.8%)
**Type of diagnosis**	
Cancer (%)	57 (28.6%)
Non-cancer (%)	142 (71.4%)

### Second assessment

For the Second Assessment, 84 patients in two groups were reassessed with PaPaS. Table 2 shows their demographics; there were no significant differences in age, gender, or diagnosis types between groups (*p* > .05).

**Table 2:** Demographics of patients at Second Assessment (*N* = 84)

Demographics	Discharged (***n*** = 21)	Continued service (***n*** = 63)
**Age at referral** ^a^		
Median age in years (IQR)	15.8 (10.3)	12.6 (11.4)
Minimum age in days	81	119
Maximum age in years	30.2	34.1
**Gender** ^b^		
Female (%)	8 (38.1%)	32 (50.8%)
Male (%)	13 (61.9%)	31 (49.2%)
**Type of diagnosis** ^c^		
Cancer (%)	0	1 (1.6%)
Non-cancer (%)	21 (100%)	62 (98.4%)

^a^ t-test found no significant difference in mean ages between the groups; *p* > .05

^b^ X^2^-test found no significant difference in gender between groups; *p* > .05

^c^ Fisher’s exact test found no significant difference in diagnosis between groups; *p* > .05

Table 3 shows the distribution of patients between groups, based on their PaPaS scores. We hypothesised that patients who scored 0–14 on the PaPaS could be considered for discharge, while those who scored 15 and above eligible for continuation of PPC. For actual patients in the ‘continued service’ group, 52 scored above 15 points, whereas all discharged patients (*n* = 21) scored less than 15. Compared against standard clinical assessment for discharge and service continuation, the PaPaS demonstrated a sensitivity of 82.54% and a specificity of 100%. Patients who continued to receive PPC after one year had a significantly higher mean score compared to patients who were discharged within one year (*p* < .001).

**Table 3:** PaPaS scores at Second Assessment (*N* = 84)

PaPaS scores (with suggested action)	Continued service (***n*** = 63)	Discharged (***n*** = 21)
**0–14 (Not for PPC)**		
Number of patients (% of column)	11 (17.46%)	21 (100%)
**15 and above (For PPC)**		
Number of patients (% of column)	52 (82.54%)	0
**Mean score** ^a^ **(SD)**	19.25 (4.74)	7.86 (2.39)

^a^ Difference of mean scores between groups = 11.39 (95% CI: 9.86–12.93); t-test: *p* < .001

## Discussion

To our knowledge, this is the first utility and feasibility study on the PaPaS—to screen patients for specialist PPC and make decisions to retain/discharge existing patients. Findings demonstrate that recommended action plans according to PaPaS scores were largely congruent with decisions made through case assessment, whether by a single clinician or MDT. Although this study was not aimed at validation, real patients’ data were used to provide substantive support for the PaPaS’s utility in identifying paediatric patients with palliative care needs [17, 18]. By comparing imputed scores with actual events in practice (i.e. clinical decisions around acceptance of new referrals), some indication of criterion validity for the PaPaS is rendered.

The PaPaS demonstrates intrinsic benefits of transparency and reliability. An objective and standardised scoring system helps to overcome variation in referral patterns among paediatricians that stem from differential interpretations of what palliative care entails [25]. The checklist approach to screening for palliative care needs is also advocated by expert consensus in the adult setting [26]. Such a tool can potentially aid policy makers in evidence-based planning, resource allocation and cost-effective commissioning of new services [27]. However, some outliers may remain that still require case-by-case consideration using alternative approaches, such as individual assessments by a clinician who is familiar with the principles of PPC.

In some settings, practitioners may find PaPaS useful for the purpose of resource allocation; to identify patients who no longer require specialist PPC after a period of intensive case management [19]. With some modifications, PaPaS demonstrates potential not only as an assessment tool for planning intake, but also discharge from and continuation of PPC. Given uncertain trajectories and huge diversity in diagnoses over wide neurodevelopmental ages, some services can consider segmentation of patients and families for long-term service sustainability. The facility for temporary discharge from PPC while the child continues to consult primary physicians in tertiary institutions is one option. The PaPaS appears to support this exercise when indicated. In fact, since late 2017, the Ministry of Health in Singapore has adopted the PaPaS to determine eligibility for home-based PPC; it is administered both on admission, and assessment for service continuation at 1 year and every year thereafter.

Another key objective in this study is to explore feasibility aspects of the instrument. In the process of imputing scores for individual patients, several ways to improve its administration were identified. These observations and suggestions are summarised in Table 4 for users’ consideration. A proposed new version of the scale is designed (Appendix) based on these comments.

**Table 4:** Domain-specific observations and comments for PaPaS for assessment of patients

Domain Item	Characteristics	Score	Comments & Recommendations (If any)
**1.1** Trajectory of disease and impact on daily activities of the child (in comparison with the child’s own baseline) (with reference to the last 4 weeks)	Stable	(0)	1. Though the two measured constructs (disease trajectory and impact on daily activities) are related, they can vary independently. Both should be scored separately. 2. Children with life-limiting conditions generally have longer disease trajectories. It is recommended that the reference baseline for Activities of Daily Living be extended to 3 months (instead of just 4 weeks) to maximise detection of change.
	Slowly deteriorating without impact on daily activities	(1)	
	Unstable; with impact on and restriction of daily activities	(2)	
	Significant deterioration with severe restriction of daily activities	(4)	
**1.2** Increase of hospital admissions, (> 50% within 3 months, compared to previous periods)	No	(0)	1. As hospital admissions are used here as a proxy for disease severity and worsening trajectory, the purpose and type of hospital stay should be considered. Elective admissions should not be included, as they could be for routine work-up (e.g. sleep studies). 2. Instead of 3 months, admissions over the last 6 months is suggested to better capture medical instability for patients with predominantly longer prognoses and variable trajectories in PPC.
	Yes	(3)	
**2.1** Treatment directed at the disease, (does not mean treatment of disease related complications, such as pain, dyspnoea or fatigue)	… is curative	(0)	1. To assess disease acuity based on *potential* treatment options, it may be helpful to qualify with an additional statement like “treatments directed at the disease, *even if not administered*”; as it was observed that many patients might not have received treatments, either due to unavailability, or they were declined by caregivers.
	… controls disease and prolongs life with good quality of life	(1)	
	… does not cure or control but has a positive effect on quality of life	(2)	
	… does not control and has no effect on quality of life	(4)	
**2.2** Burden of treatment, (Burden means side effects of treatment and additional burdens such as stay in hospital in the patient’s or family’s view)	No or minimal burden or no treatment is envisioned	(0)	1. It may be helpful to qualify further “Burden of treatments, *both disease-directed and symptom-directed*”. Treatments that are not directed at the underlying disease should also be included for consideration, as they do exact a toll on the patient and caregiver (e.g. newly ventilator-dependent) [28–32]. 2. Burden is often contingent on the intensity and caregiver skills required to administer treatment. Points of reference should be provided to ensure agreement between assessors for each level of burden (e.g. invasive treatments should be scored as high level of burden).
	Low level of burden	(1)	
	Medium level of burden	(2)	
	High level of burden	(4)	
**3.1** Symptom intensity or difficulty of symptom control (over the last 4 weeks)	Patient is asymptomatic	(0)	1. Similar to Item 1.1, the two measured constructs (severity of symptoms and difficulty in management) should be scored separately, to account for differential variations. 2. The reference baseline should be extended to “within the past 3 months”, to maximise detection of symptom related issues.
	Symptom(s) are mild and easy to control	(1)	
	Any symptom is moderate and controllable	(2)	
	Any symptom is severe or difficult to control *(unplanned hospitalisation or outpatient visits, symptom crises)*	(4)	
**3.2** Psychological distress of patient related to symptoms	Absent	(0)	1. While psychological distress is inherent in complex care [33, 34], it may be challenging to assess this accurately as these young children may struggle to articulate distress, are non-communicative, or cognitively impaired [33]. A proxy report – via either caregivers or the healthcare professional – is often required. If appropriate, this can be explicitly specified.
	Mild	(1)	
	Moderate	(2)	
	Significant	(4)	
**3.3**Psychological distress of parents or family related to symptoms and suffering of the child	Absent	(0)	1. To be accurately evaluated, psychological distress may require professional assessment. It may be difficult for most clinicians to score distress in caregivers objectively or ensure consistency between observers in that case.
	Mild	(1)	
	Moderate	(2)	
	Significant	(4)	
**5.1** Estimated life expectancy	Several years	(0)	1. There is ambiguity to the terms ‘weeks’ and ‘months’ as it is in the original scale. For example, it is unclear whether children with a prognosis of three to 4 weeks should be scored 3 or 4. Life expectancies can be better clarified using defined cut-offs, e.g. “3 months to a year”, “3 weeks to 3 months”, “less than 3 weeks”.
	Months to 1–2 years	(1)	
	Weeks to months *Please skip 5.2*	(3)	
	Days to weeks *Please skip 5.2*	(4)	
**5.2** “Would you be surprised if this child were to **suddenly** die in 6 months’ time?”	Yes	(0)	1. The word “suddenly” presents difficulty as it may be challenging to determine what changes in trajectories are considered *sudden*. It may exclude patients who die after slow deterioration, even within 6 months, if literally interpreted. Removing the term ensures that all patients who are at-risk of dying within 6 months are accounted for during scoring.
	No	(2)	

### Implications

With finite public health funding for PPC programmes, the PaPaS emerges as a tool that can systematically identify patients and families who will benefit from resource-intensive PPC at the specialist level. Widespread adoption of PaPaS may improve understanding of what PPC involves, resulting in timely referrals from paediatricians who may otherwise be unfamiliar with PPC or its service eligibility criteria [35, 36]. Current gaps in communication, continuity of care and collaboration across settings may be minimised as a consequence.

In addition, with its strength in needs assessment, delivery of existing services may be tailored based on the PaPaS to meet the ever-evolving needs of patients and families who are already receiving PPC. Our study is the first to demonstrate the potential of using PaPaS to assess the need for continuing care; it provides a transparent and objective framework to determine which patients should continue to receive PPC.

In practice however, we have encountered cases of children being ‘too well’ for palliative care, despite having obvious life-shortening conditions. It is observed in borderline cases, where patients score just under 15 points. While the use of limited resources is optimised, this is an unforeseen consequence, as a quantified tool is systematically imposed.

### Limitations

There are a number of factors that limit this study’s conclusions. First, we did not track patients who were rejected for admission or were deemed by primary physicians not to require PPC. Hence, the ability of the PaPaS to identify patients *unsuitable* for admission to PPC cannot be commented. Moreover, the study involves a retrospective review of clinical data, which did not always map specifically to items in the PaPaS. Further research can employ prospective designs where the PaPaS is used directly to assess new referrals. Second, 18 of included patients in this study were below one year of age, which was excluded in the original conceptualisation of PaPaS due to perceived differences in needs [22]. Patients beyond 19 years of age were also included in the study. As the goal was to evaluate the utility of the PaPaS in identifying eligible patients for home-based PPC as established, we had included all admitted patients, regardless of age. Third, this study did not examine inter-rater reliability. Hence, potential variability in scoring between users cannot be commented. These differences may apply to attributes that are inherently not easily quantifiable, such as psychological distress. We identified this potential issue and suggested the incorporation of cues. Still, the reliability attributes of this tool should be evaluated in future reviews. The modified scale proposed here is intended to mirror the score ranges of the original instrument as much as possible. Psychometric testing of the revised scale however, is beyond the scope of this study.

Our study findings may not be generalisable to other settings outside of home-based PPC, for example, in an in-patient palliative consult service. Similarly, perceptions of monetary concerns or other related burdens can vary in contexts with different healthcare financing systems, culture, and values. Lastly, the end-user experience should also be examined in future studies, to ensure that the PaPaS does not create additional barriers for referrals to PPC.

## Conclusion

While there are limitations in the PaPaS as an assessment tool for identifying patients with PPC needs, it demonstrates clear benefits for adoption within individual services and potentially the healthcare system. When deployed within a home hospice setting, the PaPaS has demonstrated positive qualities in terms of objectivity, transparency and accountability.

There is a tendency to over/under-estimate the need or even intensity of care, across different professionals and care settings. The PaPaS promotes greater clarity and effective handover for everyone involved, particularly at care transitions. This can lead to important outcomes like alignment of expectations between stakeholders, and critically, optimal case management.

Ultimately, the child and family living with life-shortening illness is flagged in a timely manner to receive palliative care based on needs rather than prognosis, in spite of challenges posed by disease diversity and uncertain trajectories, through a process of screening that is both robust and informational, yet with cost-containing implications.

## Data Availability

The datasets used and/or analysed during the current study are available from the corresponding author on reasonable request.

## Appendix

### Proposed revision for the Pediatric Palliative Screening scale (PaPaS) and cut-off scores*

**Table:**

Domain	Item	Characteristic	Score
Domain 1	Trajectory of disease and impact on daily activities of the child		
**1.1.1**	**With reference to the past 3 months**, the **disease trajectory** of the child, in comparison with the child’s own baseline, is…	…Stable	0 □
		…Stable, but slowly deteriorating	1 □
		…Unstable with slow deterioration	2 □
		…Unstable with significant deterioration (Please skip 1.1.2)	4 □
**1.1.2**	**With reference to the past 3 months**, the **impact of condition on daily activities** of the child, in comparison with the child’s own baseline.	No impact	0 □
		Daily activities are impacted/restricted	1 ☐
		Daily activities are severely impacted/restricted	2 □
1.2	**In the past 6 months**, there was a more than 50% Increase in **unplanned** hospital admissions (compared to previous periods)	No	0 □
		Yes	3 □
Domain 2	Expected outcome of treatment directed at the disease and burden of treatment		
2.1	Treatment directed at the disease, **even if not administered…** *(does not include treatment of disease-related complications, such as pain, dyspnea or fatigue)*	…is curative.	0 □
		…controls disease and prolongs life with good quality of life.	1 ☐
		…does not cure or control but has a positive effect on quality of life.	2 □
		…does not control and has no effect on quality of life.	4 □
2.2	Burden of treatment, **including both disease-directed and symptom-directed treatments**.*(consider frequency and skills involved;* e.g. *side effects, hospital stay, additional tasks for patients/caregivers)*	No/minimal burden OR no treatment is planned	0 □
		Low level of burden ***(*****e.g.*****simple oral medication or diet modification)***	1 □
		Medium level of burden ***(*****e.g.*****feeding tubes, catheters, medications with adverse effects)***	2 □
		High level of burden ***(*****e.g.*****hospitalization, tracheostomy, BiPAP/C-PAP, PICC line, frequent suctioning)***	4 □
Domain 3	Symptom and problem burden		
**3.1.1**	**Symptom intensity** over the past **3 months***(consider unplanned hospitalization or outpatient visits, symptom crises)*	**Patient is asymptomatic (Please skip 3.1.2)**	0 □
		Symptom(s) are mild	1 □
		Symptom(s) are moderate	2 □
		Symptom(s) are severe (Please skip 3.1.2)	4 □
**3.1.2**	**Difficulty of symptom control** over the past **3 months** *(consider unplanned hospitalization or outpatient visits, symptom crises)*	Symptom(s) are easy to control	0 □
		Symptom(s) are controllable	1 □
		Symptom(s) are difficult to control	2 □
3.2	Psychological distress of patient related to symptoms	Absent	0 □
		Mild	1 □
		Moderate	2 □
		Significant	4 □
3.3	Psychological distress of parents or family related to symptoms and suffering of the child	Absent	0 □
		Mild	1 □
		Moderate	2 □
		Significant	4 □
Domain 4	Preferences/needs of patient or parents and Preferences of health professional		
4.1	Patient/parents wish to receive palliative care or formulate needs that are best met by palliative care.	No	0 □
		Yes (Please skip 4.2)	4 □
4.2	You/your team feel that this patient would benefit from palliative care.	No	0 □
		Yes	4 □
Domain 5	Estimated life expectancy		
5.1	Estimated life expectancy/Prognosis	Several years	0 □
		1–2 years	1 □
		**3 months to a year (Please skip 5.2)**	3 □
		**Less than 3 months (Please skip 5.2)**	4 □
5.2	**Would you be surprised if this child died in 6 months’ time?**	Yes	0 □
		No	2 □
**Total Score**			

*Changes from the original scale are in bold

**Table:**

Score	Outcome during referral	Outcome during reassessment
≤ 14	Explain goals of palliative care	For discharge
≥ 15	Start palliative care	Continue with service
≥ 25	Urgent need for palliative care	Continue with service

## Abbreviations

MDT: Multi-Disciplinary Team
PaPaS: Paediatric Palliative Screening Scale
PPC: Paediatric Palliative care
